# Determinants of the intercept and slope of glomerular filtration rate in recipients of a live donor kidney transplant

**DOI:** 10.1007/s00508-020-01610-3

**Published:** 2020-02-03

**Authors:** Martina Hamböck, Anton Staudenherz, Alexander Kainz, Barbara Geist, Manfred Hecking, Konstantin Doberer, Marcus Hacker, Georg A. Böhmig

**Affiliations:** 1 Division of Nuclear Medicine, Department of Biomedical Imaging and Image-guided Therapy, Medical University of Vienna, Vienna, Austria; 2 Department of Nuclear Medicine, Molecular Imaging and Special Endocrinology, University Hospital St. Pölten—Karl Landsteiner University of Health Science, St. Pölten, Austria; 3 Division of Nephrology and Dialysis, Department of Medicine III, Medical University of Vienna, Währinger Gürtel 18–20, 1090 Vienna, Austria

**Keywords:** Antibody-mediated rejection, Donor age, Estimated glomerular filtration rate, Isotope nephrography, Kidney transplantation

## Abstract

**Background:**

Donor kidney function is considered a critical determinant of allograft survival after live donor (LD) kidney transplantation, but its independent impact on the evolution of graft function is less well defined. The objective of this study was to dissect the relative contribution of LD kidney function to baseline estimated glomerular filtration rate (eGFR) of recipients and its decline.

**Methods:**

In this study 91 LD kidney transplantations performed between 2007 and 2015 were included. The eGFR of donated kidneys (eGFR-dk) was calculated from total LD eGFR (eGFR-dt) based on the results of isotope nephrography. Recipient eGFR (eGFR-r) determined 6‑monthly until 36 months posttransplantation served as dependent variable in mixed linear models estimating changes in baseline allograft function (intercept) and eGFR‑r slope. Models were adjusted either for eGFR-dk or eGFR-dt, in addition to other potential confounders.

**Results:**

Overall, unadjusted mean eGFR‑r at baseline (6 months) and its annual decline in allograft function were 56.5 mL/min/1.73 m^2^ and −0.2 mL/min/1.73 m^2^, respectively. In multivariate analysis, eGFR-dk impacted on baseline eGFR‑r (0.6 mL/min/1.73 m^2^ mean estimated increase per unit; *P* = 0.02) but not on its slope. In the eGFR-dt-adjusted model, a marginal effect was observed for LD age (*P* = 0.05). Both models identified antibody-mediated rejection (ABMR) as the strongest risk factor of accelerated loss of allograft function (eGFR‑r slope: approximately −6 mL/min/1.73 m^2^ per year; *P* ≤ 0.02).

**Conclusion:**

Donor-related characteristics, most prominently the function of donated kidneys and LD age, were predictive of eGFR at baseline. The ABMR was identified as the cardinal cause of progressive deterioration of allograft function.

## Introduction

Live donor (LD) kidney transplantation is considered the best treatment option for patients with end-stage renal disease (ESRD), allowing superior outcomes in terms of patient survival, quality of life and health-related expenses. Careful LD selection, however, is essential to ensure best possible treatment results and maximum safety for both donors and recipients.

To facilitate the work-up of potential kidney donors, a variety of national and international guidelines have been formulated, most of them agreeing that glomerular filtration rate (GFR) should be evaluated with direct measurements of exogenous filtration markers, in addition to serum creatinine-based estimations of GFR [1, 2]. Levels of kidney function accepted for donation need to be adapted to the individual risk profile, but for individuals with a GFR of >90 mL/min per 1.73 m^2^ it is generally considered safe to donate. In addition, isotope nephrography (ING) may help to determine the relative function of the kidneys supporting the choice of the nephrectomy side [1, 2].

In recent years, there has been a trend towards the acceptance of significant LD comorbidities, especially in older individuals, provided that the lifetime risk for the development of chronic kidney disease is low [3]. This may also include donors with a GFR below the commonly accepted thresholds [4]. The use of marginal donor kidneys, however, may impact substantially on allograft performance. A prominent risk factor in this respect was shown to be LD age. Large cohort studies have revealed inferior short-term and long-term outcomes for organs originating from older donors [5–10]. Results are consistent with observations made in deceased donor (DD) kidney transplantation, where organ allocation is supported by age-matching algorithms, which take the metabolic demand of recipients into account [11].

The relative outcome effect of predonation kidney function in LD transplantation is less well studied. Norden et al. [12] observed an increased risk for graft loss in a population of 344 LD kidney transplant recipients, when donors had an unadjusted GFR below 80 mL/min. This finding is supported by a systematic review of seven studies, demonstrating associations of higher donor GFR with superior allograft function and transplant survival [13]. Definitions of GFR, however, were heterogeneous, there was no adjustment for relevant confounders, and a possible influence of unequal functional distribution between the donated and remaining kidneys was not taken into account. Moreover, none of these studies included detailed analyses of the slope of recipient estimated glomerular filtration rate (eGFR), which, confounded by a variety of immunological and non-immunological factors, may serve as a useful surrogate endpoint predicting long-term renal allograft survival [14, 15].

In this retrospective cohort study the independent impact of LD kidney function on recipient eGFR at baseline (intercept) and its slope calculated from serial eGFR measurements was investigated during the first 3 post-transplantation years. To add accuracy to the analysis the eGFR of the donated kidneys was separately calculated based on the results of Tc-99m-mercaptoacetyltriglycine acid (^99m^Tc-MAG3) scintigraphy. Mixed linear models were applied to quantify the impact of LD kidney function on allograft performance, in the context of other potentially outcome-related variables.

## Materials and methods

### Study design and patients

**Fig. 1:**
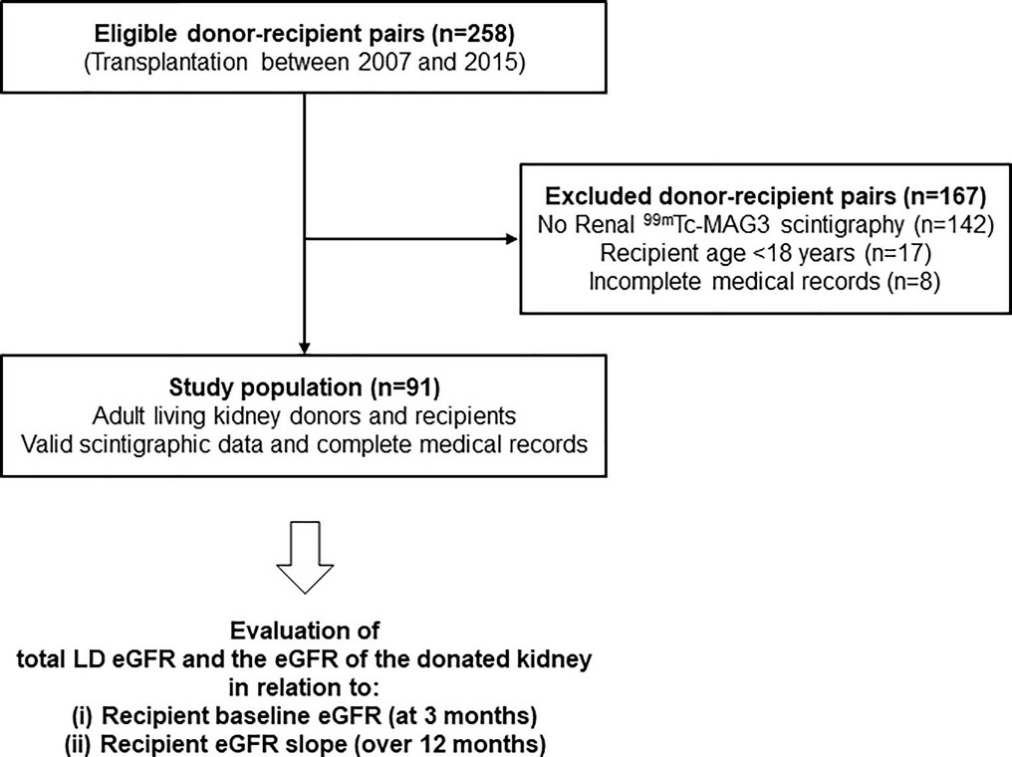
Study flow chart. Abbreviations: *eGFR* estimated glomerular filtration rate, *LD* living donor, ^*99m*^*Tc-MAG3* Tc-99m‐mercaptoacetyltriglycine acid

The primary aim of this retrospective single center cohort study was to dissect the contribution of LD kidney function, as reflected by the (i) eGFR of the donated kidney (eGFR-dk) or (ii) total donor eGFR (eGFR-dt), to baseline allograft function at 6 months (intercept) and its course until 36 months post-transplantation (slope). The study included 91 out of 258 LD allograft recipients at the Vienna transplantation unit between January 2007 and December 2015. Inclusion criteria were a recipient age of ≥18 years, the availability of ING-based split function of donated kidneys and a complete follow-up until April 2018, including serial recipient eGFR (eGFR-r) measurements at hospital discharge and at 6, 12, 18, 24 and 36 months after transplantation. Of the recipients 167 did not meet these criteria and were excluded from the analysis. A flow chart of the study is provided in Fig. 1. The study was approved by the institutional ethics committee (No. 2252/2017) and was carried out in accordance with the principles of the Declaration of Helsinki 2008 and the Declaration of Istanbul.

### Evaluation of kidney function

Estimated GFR was calculated using the Chronic Kidney Disease Epidemiology Collaboration (CKD-EPI) equation [16]. Until February 2012, 24‑h urine creatinine clearance was evaluated for LD selection. Thereafter, the donor work-up included the assessment of measured GFR (mGFR) using chromium-51 ethylenediaminetetraacetic acid (^51^Cr-EDTA). Donors received approximately 2 MBq of the radiolabelled filtration marker, and sequentially drawn blood samples (120, 180 and 240 min after administration) served to determine the plasma clearance. Body surface area-adjusted GFR values were calculated with in-house software, as described by Geist et al. [17]. According to our local standard, a creatinine clearance or an adjusted mGFR below 80 mL/min was considered to be a contraindication for donor nephrectomy.

### Isotope nephrography

Renal ^99m^Tc-MAG3 scintigraphy to determine the relative functional distribution between the two donor kidneys (split kidney function) was performed according to the protocol of the European Association of Nuclear Medicine [18]. Image acquisition was performed with a gamma camera, as previously described [19]. The imaging software HERMES Gold^TM^ (Hermes Medical Solutions AB, Stockholm, Sweden) was used to draw regions of interest (ROIs) around the kidneys, the heart and the perirenal background. The mean transit time (MTT) and the relative kidney function from 1 min to 3 min were extracted from the integrals of renal time activity curves (TACs). The LD candidates with a side difference of >20% (>60% vs. <40%) were not accepted for donation. The relative function determined by renal MAG3 scintigraphy was used to calculate eGFR-dk and the mGFR of the donated kidney (mGFR-dk) by its multiplication with eGFR-dt or total LD mGFR (mGFR-dt), respectively. The eGFR of the remaining kidney (eGFR-rk) was calculated by subtraction of eGFR-dk from eGFR-dt. MTT values of 1.9–2.9 min were considered normal [20].

### Immunosuppression

The majority of included recipients (89%) received calcineurin inhibitor-based maintenance immunosuppression, commonly triple therapy including tacrolimus, mycophenolic acid and steroids (Table 1). Most recipients (90%) also received interleukin (IL)-2 receptor antibody induction. During follow-up maintenance immunosuppression was changed in 14 of the patients (tacrolimus to cyclosporin A: *n* = 4; tacrolimus to sirolimus or everolimus: *n* = 4; belatacept to tacrolimus: *n* = 3; sirolimus or everolimus to tacrolimus: *n* = 2; cyclosporin A to tacrolimus: *n* = 1). Median tacrolimus trough levels were 7.7 ng/mL and 6.3 ng/mL after 6 and 12 months, respectively. Of the patients eight were transplanted across major ABO barriers, following a course of ABO antigen-specific (*n* = 6) or semi-selective (in cases of additional preformed anti-HLA donor-specific antibodies, DSA: *n* = 2) immunoadsorption and a single dose of rituximab and intravenous immunoglobulin (IVIG).

**Table 1:** Recipient baseline characteristics

Parameters	Total (*n* = 91)
*Variables recorded at the time of transplantation*	
Age, years, median (IQR)	42.4 (28.1–54.1)
Female sex, *n* (%)	32 (35.2)
Underlying kidney disease, *n* (%)	
Glomerulonephritis	39 (42.9)
Polycystic kidney disease	18 (19.8)
Obstructive nephropathy	11 (12.1)
Diabetic nephropathy	3 (3.3)
Hypertensive nephropathy	1 (1.1)
Other, unknown	19 (20.9)
Preemptive transplantation, *n* (%)	28 (30.8)
Recipient of a retransplant, *n* (%)	10 (11)
ABO/HLA-incompatible transplantation, *n* (%)^a^	10 (11)
Sum of HLA mismatch in A, B and DR, median (IQR)	3 (2–4)
Baseline immunosuppression, *n* (%)	
Induction with IL-2R antibody	82 (90.1)
Tacrolimus	75 (82.4)
Trough level at 6 months, ng/ml, median (IQR)	7.7 (6.0–9.1)
Trough level at 12 months, ng/ml, median (IQR)	6.3 (5.2–8.0)
Cyclosporin A	6 (6.6)
mTOR inhibitor^b^	3 (3.3)
Belatacept	6 (6.6)
Mycophenolic acid	88 (96.7)
Azathioprine	1 (1.1)
Steroid	90 (98.9)

*IL-2R* interleukin‑2 receptor, *IQR* interquartile range, *mTOR* mammalian target of rapamycin

^a^Eight patients were transplanted across ABO and two patients across both ABO and HLA-donor-specific antibody (DSA) barriers

^b^Sirolimus or everolimus

### Transplant biopsies

Indication biopsies were performed for graft dysfunction and/or significant proteinuria. Our standard did not include surveillance biopsies. Histomorphology and immunohistochemistry were evaluated on formalin-fixed paraffin-embedded sections. T cell-mediated rejection (TCMR) and antibody-mediated rejection (ABMR) were defined according to the 2015 update of the Banff classification of renal allograft pathology [21].

### Statistical analysis

Continuous data were expressed as median and interquartile range (IQR) and categorical variables as absolute and relative frequencies. Kaplan-Meier analysis was applied for calculation of graft and patient survival. The influence of LD kidney function on baseline eGFR‑r and on its slope was evaluated using mixed linear models. We calculated two separate models, in which LD kidney function was either characterized by eGFR-dk or by eGFR-dt and LD kidney function and time were included in each calculation. Slope estimates additionally considered interactions of variables with time. In the reduced model, several other donor and recipient variables were added one by one. The multivariable model was expanded by variables with a *P* value of <0.157 for their impact on baseline eGFR‑r or its slope in the reduced model [22]. Levels of eGFR‑r from 6 months to 36 months were used as dependent variables. For correlation analysis, Spearman’s rank correlation test was applied. A 2-sided *P* < 0.05 was considered significant. For statistical analysis, IBM SPSS Statistics 23 for Mac (IBM Corporation, Armonk, NY, USA) and SAS 9.4 for Windows (The SAS Institute Inc., Cary, NC, USA) were used.

## Results

### Patient characteristics

The study included 91 adult recipients of a LD kidney allograft. Key inclusion criteria were a detailed ING-based LD work-up and a complete recipient follow-up.

Baseline donor and recipient data are provided in Tables 1 and 2, respectively. The median recipient age was 42 years and 35% of the patients were female. The most common causes of ESRD were glomerulonephritis and polycystic kidney disease, 31% of the patients underwent preemptive transplantation and 11% were recipients of a re-transplant. The median sum of HLA mismatches in A, B and DR was three (Table 1).

**Table 2:** Results of LD kidney evaluation

Parameters	Total (*n* = 91)
*Variables recorded at the time of donation*	
Age, years, median (IQR)	51.6 (44.2–57.2)
Female sex, *n* (%)	57 (62.6)
BMI, kg/m^2^, median (IQR)	25.6 (22.9–28.7)
Living-related, *n* (%)	53 (58.2)
Donation of left kidney, *n* (%)	73 (80.2)
Total eGFR (eGFR-dt), mL/min/1.73 m^2^, median (IQR)	87 (77–98)
Total mGFR (mGFR-dt), mL/min/1.73 m^2^, median (IQR)^a^	120 (104–139)
ING-based parameters of donated kidney, median (IQR)	
Mean transit time (MTT), min	2.9 (2.6–3.3)
Relative function, %	51 (48–54)
eGFR according to relative function (eGFR-dk)^b^, mL/min/1.73 m^2^	43 (38–50)
mGFR according to relative function (mGFR-dk)^a,b^, mL/min/1.73 m^2^	62 (51–71)

*BMI* body mass index, *eGFR* estimated glomerular filtration rate, *ING* isotope nephrography, *IQR* interquartile range, *LD* live donor, *mGFR* measured glomerular filtration rate

^a^mGFR measurements were available for 53 donors

^b^eGFR-dk and mGFR-dk were calculated from eGFR-dt or mGFR-dt on the basis of the relative kidney function determined in ING

The LD were in median 52 years old, and 63% were female and 53% of the donors were living-related. Evaluation of LD kidney function revealed a median eGFR-dt of 87 mL/min/1.73 m^2^ and a median mGFR-dt of 120 mL/min/1.73 m^2^, respectively. The ING-based analysis revealed a median MTT of 2.9 min and a median relative organ function of 51% for the donated kidneys, of which 80.2% were left kidneys. Median eGFR-dk and mGFR-dk were 43 and 62 mL/min/1.73 m^2^, respectively (Table 2).

### Allograft and recipient outcomes

**Fig. 2:**
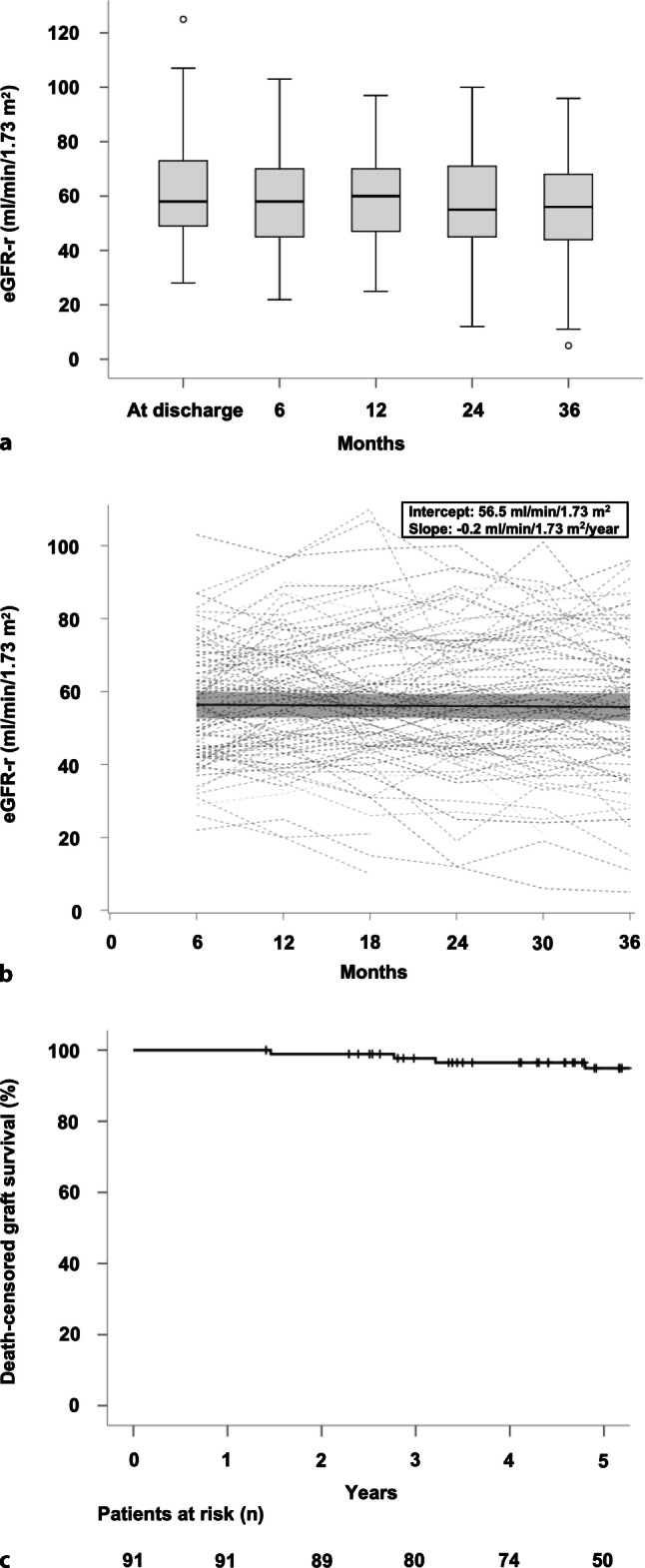
Transplant and patient outcomes. **a** Boxplots indicate the median, interquartile range, minimum and maximum of recipient estimated glomerular filtration rate (eGFR-r) at hospital discharge and at 6, 12, 18, 24 and 36 months after transplantation. **b** Individual courses of eGFR‑r (*dashed lines*) as well as its estimated mean (*solid line*) and the 95% confidence interval (*grey area*) computed from an unadjusted mixed model are shown for a period between 6 months (intercept) and 3 years after transplantation. **c** Kaplan-Meier curves show death-censored graft survival over a period of 5 years

Transplant outcomes are detailed in Table 3. The course of eGFR‑r until 36 months posttransplantation is illustrated in Fig. 2. In the overall cohort, the unadjusted mean baseline eGFR‑r at 6 months (intercept) was 56.5mL/min/1.73 m^2^ (95% CI: 52.3–60.7mL/min/1.73 m^2^), and the unadjusted mean annual decline in allograft function (slope) was −0.2 (−1.8–1.3) mL/min/1.73 m^2^.

**Table 3:** Transplant outcomes

Parameters	Total (*n* = 91)
*Variables recorded after transplantation*	
Recipient eGFR (eGFR-r), mL/min/1.73 m^2^, median (IQR)^a^	
At discharge^b^	57 (46–72)
6 months	56 (44–67)
12 months	60 (47–70)
24 months	55 (45–71)
36 months	55 (44–67)
Histopathological findings in indication biopsies, *n* (%)	
TCMR	18 (19.8)
ABMR	10 (11)
De novo/recurrent glomerulonephritis	4 (4.4)
BK virus nephropathy	3 (3.3)
Death-censored graft survival, %^c^	
1 year	100
3 years	98
5 years	95
Patient survival, %	
1 year	100
3 years	98
5 years	98

*ABMR* antibody-mediated rejection, *eGFR* estimated glomerular filtration rate, *IQR* interquartile range, *TCMR* T cell-mediated rejection

^a^Patients who lost their graft were assigned an eGFR of 0 mL/min/1.73 m^2^

^b^eGFR was recorded at the day of hospital discharge after kidney transplantation

^c^Causes of graft loss: ABMR (*n* = 6), BK virus nephropathy (*n* = 1), unknown (*n* = 2)

**Fig. 3:**
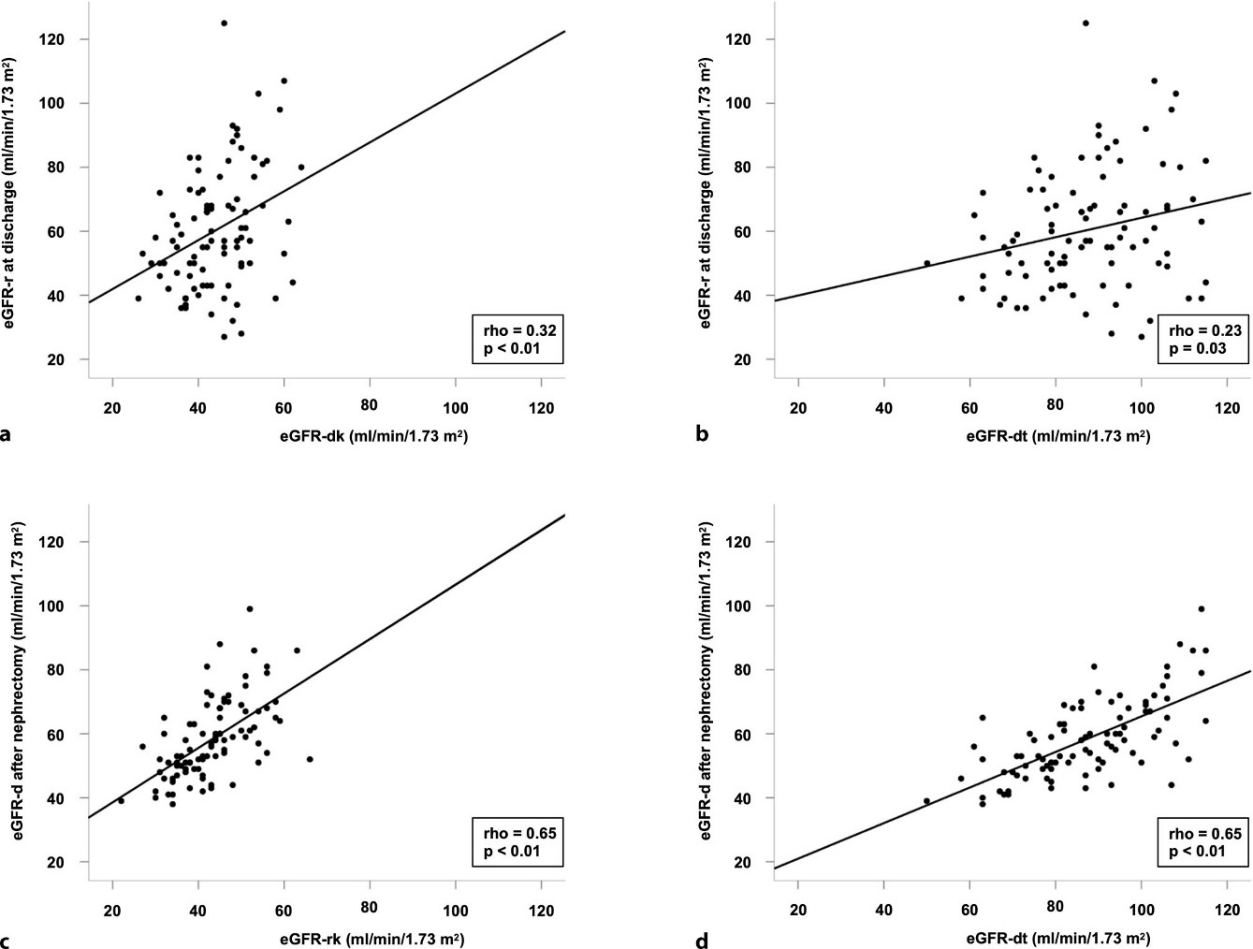
Correlations between **a** predonation estimated glomerular filtration (eGFR) of the donated kidney (eGFR-dk) and recipient eGFR (eGFR-r) at discharge, **b** predonation total donor eGFR (eGFR-dt) and eGFR‑r at discharge, **c** eGFR of the remaining donor kidney (eGFR-rk) and donor eGFR (eGFR-d) 1 week after nephrectomy, as well as **d** predonation eGFR-dt and eGFR‑d 1 week after nephrectomy. Data are visualized by scatter plots and corresponding regression lines (*solid lines*) demonstrating the correlations between donor and recipient eGFR values. For statistical evaluation, test results were compared using Spearman’s rank correlation analysis

Levels of eGFR‑r at hospital discharge correlated with predonation LD eGFR (Fig. 3). Correlations were stronger if donor kidney function was characterized by the eGFR-dk than the eGFR-dt (rho = 0.32 versus rho = 0.23). Moreover, there was a close correlation between predonation LD eGFR, expressed as eGFR-dt or eGFR of the remaining kidney (eGFR-rk), and postdonation LD eGFR (rho = 0.65) (Fig. 3).

The most common histopathological findings in indication biopsies were TCMR (*n* = 18) and ABMR (*n* = 10). Following the Banff 2015 scheme, 3 recipients were diagnosed with acute active ABMR, and 7 recipients with chronic active ABMR (Table 3).

The 1‑year, 3‑year and 5‑year death-censored graft survival rates were 100%, 98% and 95%, respectively (Fig. 2). Of the patients 9 lost their transplants after a median interval of 5.7 years, most commonly (6 cases) as a result of ABMR (BK virus nephropathy: *n* = 1; unknown cause: *n* = 2). Patient survival at 1, 3 and 5 years was 100%, 98% and 98%, respectively (Table 3). Overall, three deaths were recorded during follow-up (two with a functioning allograft).

### Effect of donor kidney function on recipient eGFR

We applied two separate mixed linear models to characterize the effects of LD kidney function on eGFR‑r. The first model (Table 4) was adjusted for eGFR-dk and other relevant donor- or recipient-related variables. Multivariable analysis revealed a significant impact of eGFR-dk on eGFR‑r at baseline (0.6mL/min/1.73 m^2^, 95% CI: 0.1–1.1mL/min/1.73 m^2^ mean estimated increase per unit; *P* = 0.02) but not on eGFR‑r slope (*P* = 0.27). The ABMR was the strongest predictor of eGFR‑r slope (mean estimated annual decline: −5.8 (−10.4 to −1.2) mL/min/1.73 m^2^; *P* = 0.01). We also observed a marginal effect of donor body mass index (BMI; *P* = 0.04). Other variables selected for multivariate analysis, including LD age, donor and recipient gender, baseline immunosuppression or MTT, however, had no significant effect. Notably, also pre-emptive transplantation was not associated with allograft function.

**Table 4:** Mixed linear model to evaluate the impact of the function of the donated kidney on recipient eGFR

Parameters	Reduced model^a^		Multivariable model^b^	
	Estimate (95% CI)	*P* value	Estimate (95% CI)	*P* value
*Impact on recipient baseline eGFR, mL/min/1.73 m*^*2*^				
eGFR-dk^c^, per mL/min/1.73 m^2^	Changes with each calculation		0.6 (0.1 to 1.1)	0.02
Recipient variables				
Age, per year	−0.2 (−0.5 to 0)	0.06	−0.2 (−0.4 to 0.1)	0.23
Female sex, yes vs. no	3.5 (−4.7 to 11.7)	0.40	–	–
Preemptive transplantation, yes vs. no	1.9 (−6.4 to 10.3)	0.65	–	–
Recipient of a re-transplant, yes vs. no	4.7 (−8 to 17.3)	0.46	–	–
ABO/HLA incompatible transplantation, yes vs. no	1.3 (−11.2 to 13.9)	0.83	–	-
Sum of HLA mismatch in A, B and DR, per *n*	−0.9 (−3.5 to 1.8)	0.52	–	–
IL-2R antibody induction, yes vs. no	−7.5 (−20.5 to 5.5)	0.26	–	–
Tacrolimus-based immunosuppression, yes vs. no	2.6 (−7.5 to 12.6)	0.61	–	–
Belatacept-based immunosuppression, yes vs. no	−0.3 (−15.8 to 15.3)	0.97	–	–
LD variables				
Age, per year	−0.5 (−0.9 to 0)	0.04	−0.4 (−0.9 to 0)	0.08
Female sex, yes vs. no	−6.4 (−14.5 to 1.8)	0.12	−3.8 (−12.0 to 4.3)	0.35
BMI, per kg/m^2^	0.8 (−0.2 to 1.8)	0.133	0.6 (−0.4–1.6)	0.24
Living-related, yes vs. no	−2.5 (−10.3 to 5.4)	0.53	–	–
Abnormal MTT of donated kidney^d^, yes vs. no	−6.8 (−14.4 to 0.9)	0.08	−6.2 (−13.5 to 1.2)	0.10
Biopsy-proven rejection				
TCMR, yes vs. no	0.1 (−9.5 to 9.7)	0.99	−2.0 (−11.3 to 7.2)	0.66
ABMR, yes vs. no	0.5 (−11.9 to 12.8)	0.94	1.6 (−10.1 to 133)	0.79
*Impact on recipient eGFR slope, mL/min/1.73 m*^*2*^*/year*				
eGFR-dk^c^, per mL/min/1.73 m^2^	Changes with each calculation		−0.1 (−0.3 to 0.1)	0.27
Recipient variables				
Age, per year	0.1 (0 to 0.2)	0.26	0 (−0.1 to 0.1)	0.39
Female sex, yes vs. no	−2.1 (−5.2 to 1.1)	0.20	–	–
Preemptive transplantation, yes vs. no	1.1 (−2.1 to 4.4)	0.49	–	–
Recipient of a re-transplant, yes vs. no	0.9 (−4.2 to 6)	0.73	–	–
ABO/HLA incompatible transplantation, yes vs. no	−0.5 (−5.4 to 4.4)	0.85	–	–
Sum of HLA mismatch in A, B and DR, per *n*	0.3 (−0.8 to 1.3)	0.59	–	–
IL-2R antibody induction, yes vs. no	1.4 (−3.5 to 6.3)	0.58	–	–
Tacrolimus-based immunosuppression, yes vs. no	2.5 (−1.4 to 6.4)	0.20	–	–
Belatacept-based immunosuppression, yes vs. no	−0.9 (−6.7 to 4.9)	0.77	–	–
LD variables				
Age, per year	−0.2 (−0.3 to 0)	0.09	−0.2 (−0.4 to 0)	0.07
Female sex, yes vs. no	2.5 (−0.6 to 5.7)	0.11	0.9 (−2.4 to 4.1)	0.60
BMI, per kg/m^2^	−0.4 (−0.8 to −0.01)	0.03	−0.5 (−0.8 to −0)	0.04
Living-related, yes vs. no	−0.2 (−3.3 to 2.8)	0.87	–	–
Abnormal MTT of donated kidney^d^, yes vs. no	0.6 (−2.4 to 3.6)	0.71	0.9 (−2.1 to 3.8)	0.56
Biopsy-proven rejection				
TCMR, yes vs. no	−2.8 (−6.5 to 0.9)	0.13	−1.2 (−4.9 to 2.5)	0.51
ABMR, yes vs. no	−5.3 (−10 to −0.6)	0.03	−5.8 (−10.4 to −1.2)	0.01

*ABMR* antibody-mediated rejection, *BMI* body mass index, *CI* confidence interval, *eGFR* estimated glomerular filtration rate, *eGFR-dk* eGFR of the donated kidney, *IL-2R* interleukin‑2 receptor, *LD* live donor, *MTT* mean transit time, *TCMR* T cell-mediated rejection

^a^In the reduced model recipient eGFR values from 6 to 36 months were used as dependent variable. eGFR-dk and time were included in each calculation. Other variables were added one by one. Slope estimates additionally considered interactions of variables with time

^b^The multivariable model included eGFR-dk and time. Additionally, the model was expanded by variables with a *P* value of <0.157 for their impact on recipient baseline eGFR or its slope in the reduced model

^c^eGFR-dk was calculated from total donor eGFR on the basis of the relative kidney function determined in isotope nephrography

^d^MTT values of 1.9 to 2.9 min were considered normal

The second model (Table 5) included the same variables but was adjusted for eGFR-dt. There was no significant effect of eGFR-dt on eGFR‑r at baseline (*P* = 0.14) or its slope (*P* = 0.52). In this model, however, increasing LD age showed a marginal association with lower baseline eGFR‑r (−0.5 (−1 to 0) mL/min/1.73 m^2^ mean estimated decrease per year; *P* = 0.05). As in the first model, ABMR occurrence had a strong impact on eGFR‑r slope (mean estimated annual decline: −5.7 (−10.4 to −1.0) mL/min/1.73 m^2^; *P* = 0.02). In this model, only a slight effect was observed for BMI (*P* = 0.05).

**Table 5:** Mixed linear model to evaluate the impact of total LD kidney function on recipient eGFR

Parameters	Reduced model^a^		Multivariable model^b^	
	Estimate (95% CI)	*P* value	Estimate (95% CI)	*P* value
*Impact on recipient baseline eGFR, mL/min/1.73* *m*^*2*^				
eGFR-dt, per mL/min/1.73 m^2^	Changes with each calculation		0.2 (−0.1 to 0.5)	0.14
Recipient variables				
Age, per year	−0.3 (−0.5 to 0)	0.04	−0.2 (−0.4 to 0.1)	0.20
Female sex, yes vs. no	4.8 (−3.4 to 13.1)	0.25	–	–
Preemptive transplantation, yes vs. no	1.9 (−6.5 to 10.4)	0.65	–	–
Recipient of a re-transplant, yes vs. no	6.4 (−6.4 to 19.1)	0.32	–	–
ABO/HLA incompatible transplantation, yes vs. no	0.2 (−12.5 to 13)	0.97	–	–
Sum of HLA mismatch in A, B and DR, per *n*	−0.7 (−3.4 to 2)	0.60	–	–
IL-2R antibody induction, yes vs. no	−7 (−20.2 to 6.2)	0.29	–	–
Tacrolimus-based immunosuppression, yes vs. no	1.3 (−8.9 to 11.5)	0.80	–	–
Belatacept-based immunosuppression, yes vs. no	−0.1 (−15.8 to 15.7)	0.99	–	–
LD variables				
Age, per year	−0.6 (−1.1 to −0.1)	0.02	−0.5 (−1 to 0)	0.05
Female sex, yes vs. no	−7.1 (−15.3 to 1.1)	0.09	−4.6 (−12.8 to 3.7)	0.28
BMI, per kg/m^2^	0.72 (−0.3 to 1.7)	0.16	0.5 (−0.5 to 1.5)	0.30
Living-related, yes vs. no	−3.2 (−11.2 to 4.7)	0.42	–	–
Abnormal MTT of donated kidney^c^, yes vs. no	−8.3 (−16 to −0.5)	0.04	−6.6 (−14.2 to 0.9)	0.08
Biopsy-proven rejection				
TCMR, yes vs. no	1.4 (−8.4 to 11.3)	0.77	−1.3 (−10.7 to 8.1)	0.78
ABMR, yes vs. no	0.8 (−11.9 to 13.5)	0.90	1.4 (−10.6 to 13.4)	0.81
*Impact on recipient eGFR slope, mL/min/1.73* *m*^*2*^*/year*				
eGFR-dt, per mL/min/1.73 m^2^	Changes with each calculation		0 (−0.1 to 0.1)	0.52
Recipient variables				
Age, per year	0.1 (0 to 0.2)	0.21	0 (−0.1 to 0.1)	0.20
Female sex, yes vs. no	−2.2 (−5.3 to 1)	0.17	–	–
Preemptive transplantation, yes vs. no	1.3 (−2 to 4.5)	0.44	–	–
Recipient of a re-transplant, yes vs. no	0.5 (−4.6 to 5.6)	0.84	–	–
ABO/HLA incompatible transplantation, yes vs. no	−0.5 (−5.5 to 4.4)	0.84	–	–
Sum of HLA mismatch in A, B and DR, per *n*	0.3 (−0.8 to 1.3)	0.60	–	–
IL-2R antibody induction, yes vs. no	1.1 (−3.8 to 6.1)	0.65	–	–
Tacrolimus-based immunosuppression, yes vs. no	2.6 (−1.3 to 6.5)	0.19	–	–
Belatacept-based immunosuppression, yes vs. no	−1 (−6.8 to 4.8)	0.74	–	–
LD variables				
Age, per year	−0.1 (−0.3 to 0.1)	0.18	−0.2 (−0.4 to 0)	0.11
Female sex, yes vs. no	2.7 (−0.4 to 5.9)	0.09	1.0 (−2.2 to 4.3)	0.53
BMI, per kg/m^2^	−0.4 (−0.8 to 0)	0.03	−0.4 (−0.8 to 0)	0.05
Living-related, yes vs. no	−0.3 (−3.3 to 2.8)	0.87	–	–
Abnormal MTT of donated kidney^c^, yes vs. no	0.6 (−2.4 to 3.6)	0.70	1.0 (−2.0 to 4.0)	0.53
Biopsy-proven rejection				
TCMR, yes vs. no	−2.7 (−6.5 to 1)	0.15	−1.4 (−5.1 to 2.4)	0.47
ABMR, yes vs. no	−5.1 (−9.8 to −0.3)	0.04	−5.7 (−10.4 to −1.0)	0.02

*ABMR* antibody-mediated rejection, *BMI* body mass index, *CI* confidence interval, *eGFR* estimated glomerular filtration rate, *eGFR-dt* total donor eGFR, *IL-2R* interleukin‑2 receptor, *LD* live donor, *MTT* mean transit time, *TCMR* T cell-mediated rejection

^a^In the reduced model recipient eGFR values from 6 to 36 months were used as dependent variable. eGFR-dt and time were included in each calculation. Other variables were added one by one. Slope estimates additionally considered interactions of variables with time

^b^The multivariable model included eGFR-dt and time. Additionally, the model was expanded by variables with a *P* value of <0.157 for their impact on recipient baseline eGFR or its slope in the reduced model

^c^MTT values of 1.9–2.9 min were considered normal

## Discussion

The primary objective of this study was to dissect the relative impact of LD kidney function on recipient baseline eGFR (intercept) at 6 months and eGFR slope. Major results of multivariable analysis were that the eGFR of donated kidneys, and in addition LD age, had an independent effect on allograft function at baseline, while there was no meaningful effect on eGFR slope. In line with earlier studies [23–25], ABMR was the dominant cause of transplant functional decline, with an associated mean eGFR slope of approximately −6 mL/min/1.73 m^2^ per year as compared to −0.2 mL/min/1.73 m^2^ per year in the overall cohort.

LD kidney transplantation is the best available treatment option for patients with ESRD, allowing for excellent clinical outcomes, with 1‑ and 5‑year graft survival rates of 96% and 87%, respectively shown for Europe [26]. Due to substantial demographical changes, however, the demand for donor organs is constantly rising. In recent years, there has been a progressive increase in the use of older LD, who often present with additional risk factors, such as obesity, hypertension or subnormal GFR levels (even below 60 mL/min/1.73 m^2^) [4]. A trend towards the use of marginal donors raises major safety concerns regarding long-term LD outcomes. In addition, such variables, most prominently LD age and renal function, may also be important independent correlates of recipient allograft function. Previous studies have shown that recipients of kidneys from older donors are at an increased risk of delayed graft function, graft failure and death [5, 6]. Similar associations have been observed for subnormal predonation GFR, but in smaller recipient cohorts and in less well-designed studies [13].

For our study, we have chosen baseline eGFR‑r at 6 months and eGFR‑r slope calculated from 6‑monthly measurements as dependent variables in mixed linear models. There is increasing evidence that the extent of eGFR decline over time may serve as a valuable surrogate endpoint for long-term renal survival, in both transplantation [14, 15] and native kidney disease [27, 28]. For example, evaluating a consecutive cohort of 508 non-sensitized DD or LD renal allograft recipients, Wiebe et al. [23] described a close interrelationship between eGFR and long-term graft survival. Focusing on a specific subgroup of renal allograft recipients who developed de novo donor-specific antibodies (*dn*DSA), a highly significant 6% increase in the risk of post-*dn*DSA graft loss was calculated for each 1 mL/min/1.73 m^2^ decease in eGFR at 3 years postsubclinical *dn*DSA onset [23].

We identified donor kidney function and, in accordance with previous studies [9, 10], donor age as independent predictors of baseline eGFR‑r, reinforcing the usefulness of these parameters for risk stratification of organs from potential kidney donors. In our cohort of LD kidney transplants, eGFR-dk was associated with a mean estimated increase in recipient baseline eGFR of 0.6 mL/min/1.73 m^2^ per unit and increasing donor age was associated with a marginal decrease in recipient baseline eGFR. In contrast, we found no significant effect of total kidney function in the eGFR-dt-adjusted model. This result indicates a diagnostic benefit of ING for assessment of functional side distribution in the context of LD evaluation; however, we are aware of the limited sample size which may have precluded detection of subtle differences. For another ING-based parameter—MTT to quantify the dynamics of parenchymal tracer transit—we found no association with any of the endpoints, suggesting that this parameter may have a limited diagnostic value in the evaluation of normal functioning kidneys; however, impaired renal transit may help to dissect certain disease states, such as acute tubular injury or cyclosporine toxicity in renal transplants [29].

Interestingly, while there was a marginal effect of BMI, our study did not reveal a significant effect of LD kidney function (and age) on the slope of eGFR‑r. This finding was unexpected considering the potential functional impact of a limited renal functional reserve associated with lower donor GFR, which may ultimately cause injury due to hyperfiltration in remaining nephrons [30]; however, we want to point out that our local standard did not accept donors with an adjusted measured GFR (or a urinary creatinine clearance) <80 mL/min and/or an unequal distribution of kidney function detected by ING (>20% side difference), and this policy resulted in the overall inclusion of donated kidneys with favorable baseline function (median eGFR-dk: 43 (IQR: 38–50) mL/min/1.73 m^2^; median relative function: 51 (48–54) %). Our results are in line with an earlier analysis of 4488 patients, mostly DD recipients, where donor age had a significant impact on recipient eGFR at 12 months, but no influence on eGFR slope [9]. Perhaps as a result of inherent differences in case selection, which may also include marked differences in donor characteristics, other studies have revealed controversial results. For example, in a study by Issa et al. [8] changes in eGFR of LD kidney recipients over a period of 2 years after transplantation were estimated at −8.76 mL/min/1.73 m^2^, if donors were aged ≥45 years and at −7.40 mL/min/1.73 m^2^, if donors had an unadjusted predonation eGFR of <110 mL/min. Moreover, also in two other larger studies [7, 10], donor age was reported to be a significant determinant of progressive functional deterioration of renal allografts, in one of these studies [10], however, only beyond the first post-transplantation year.

A major finding of our study was that ABMR (10 recipients in our cohort) turned out to be the strongest predictor of annual eGFR‑r decline. The diagnosis of acute or chronic active ABMR, the leading cause of graft failure in our cohort (six of nine recorded allograft losses), was found to be associated with a mean eGFR‑r slope of approximately −6 mL/min/1.73 m^2^ per year. This observation is consistent with the previous literature reinforcing a deleterious impact of ABMR on kidney allograft outcomes [31]. Few studies have analyzed the finding of *dn*DSA or diagnosis of ABMR in relation to the dynamics of eGFR decline. For example, Wiebe et al. [23] found an eGFR decline of −3.15 and −5.61 mL/min/1.73 m^2^ per year in patients with subclinical (*n* = 19) and clinical (*n* = 45) *dn*DSA, respectively. Moreover, in a recent randomized controlled trial evaluating bortezomib in 44 subjects with late ABMR, eGFR slopes were about −5 mL/min/1.73 m^2^ per year in both placebo and treatment groups [24]. Similar results (eGFR slope of approximately −7 mL/min/1.73 m^2^ per year among 25 randomized subjects) were reported in a trial evaluating the effect of combined IVIG and rituximab in ABMR with transplant glomerulopathy [25]. The unfavorable course of allograft function in patients with ABMR, as opposed to TCMR, may reflect the current unavailability of effective therapeutic measures to counteract this type of rejection, in particular late ABMR associated with chronic irreversible injury [24, 25]. Our data reinforce the need for the establishment of effective measures to prevent or treat ABMR.

Our study has several inherent limitations. One major limitation is the comparatively small sample size, which was due to the monocentric study design and limited availability of ING data in our cohort. While we were able to dissect strong independent predictors of graft function evolution, our study may have not been sufficiently powered to detect subtle effects of some other potentially confounding variables, such as baseline immunosuppression (e.g. calcineurin inhibitors versus belatacept, which may delay progressive functional deterioration [32]). Another limitation is the intermediate-term follow-up (median 7 years), which in our cohort of LD kidney transplant recipients coincided with a low rate of graft loss (10%). Therefore, based on previous studies, we have selected eGFR slope as a surrogate endpoint, which allowed us to detect relevant outcome differences even in (i) a smaller cohort and (ii) after a shorter follow-up period. Finally, it may also be considered a limitation that our analysis was based on serum creatinine-based estimations of donor kidney function. Measured GFR was available only for half of the included LD and the resulting sample size would have been too small to detect meaningful effects. For our study, we have chosen the CKD-EPI equation, which, in contrast to other equations, such as the MDRD equation, may more accurately reflect the GFR in subjects with normal renal function [16].

While our results support that LD kidney function and age independently predict allograft function at baseline, we were not able to demonstrate a significant effect of these variables on the slope of recipient eGFR. In contrast, occurrence of ABMR turned out to be the strongest risk factor for accelerated loss of allograft function after LD kidney transplantation.
